# Upregulated circTMEM59 Inhibits Cell Growth and Metastasis by miR-668-3p/ID4 Axis in Colorectal Cancer

**DOI:** 10.1155/2022/7242124

**Published:** 2022-05-24

**Authors:** Yifei Feng, Xiaoyuan Wang, Changzhi Huang, Dongsheng Zhang, Tingwei Liu, Chuan Zhang, Yue Zhang, Dongjian Ji, Junwei Tang, Yueming Sun

**Affiliations:** ^1^Department of General Surgery, The First Affiliated Hospital of Nanjing Medical University, Nanjing, China; ^2^The First School of Clinical Medicine, Nanjing Medical University, Nanjing, China; ^3^Nanjing Medical University, Nanjing, China; ^4^Department of General Surgery, The Second Affiliated Hospital of Nanjing Medical University, Nanjing, China

## Abstract

The incidence and mortality of colorectal cancer (CRC) are ranked in the top three worldwide in 2020. Abundant studies have reported that circular RNAs (circRNAs) act critical roles in the genesis and development of tumors, including CRC. Nevertheless, the roles and detailed regulation mechanisms of circRNAs that are related to the initiation and development of CRC have not been fully found and clarified. This research primarily revealed that circTMEM59 was greatly downregulated in CRC tissues and cell lines via qRT-PCR. In addition, the decreased expression of circTMEM59 was closely related to adverse clinicopathological characteristics and the shorter survival time of CRC patients. Then, a further study found that the overexpression of circTMEM59 suppressed cell growth and accelerated the cell death of CRC via a series of experiments in vitro and in vivo. Furthermore, circTMEM59 also repressed the metastatic behaviors of CRC cells. Further study revealed that circTMEM59 played the role of competing endogenous RNAs (ceRNAs) by binding to miR-668-3p to increase the expression of inhibitor of DNA binding 4 (ID4) in CRC. In summary, the results of this study clarified the antitumor effects of circTMEM59/miR-668-3p/ID4 axis in CRC progression and provided potential therapeutic targets and clinical prognostic markers for CRC.

## 1. Introduction

According to the report of Global Cancer Statistics 2020, colorectal cancer (CRC) is the most common gastrointestinal malignant tumor worldwide. Its incidence (about 188 million new cases) and mortality (about 91 million cases) are among the top three of all malignant tumors [1]. Approximately 555,000 new CRC patients and 286,000 deaths in China in 2020 are a burden to the whole society [2]. Although the survival rate of CRC patients greatly improved with the advances of comprehensive treatment programs, such as neoadjuvant therapy, radical surgery, postoperative radiotherapy, postoperative adjuvant chemotherapy, and targeting immunotherapy, the five-year survival rate of CRC patients with distant metastasis is only 14% [3–5]. Therefore, to improving the detection rate of early CRC patients [6], it is of great clinical significance and value to explore the detailed molecular mechanisms in the pathogenesis and development of CRC to strengthen the therapeutic effects on CRC patients.

Circular RNAs (circRNAs), which are noncoding RNAs with a loop structure that are more stable than linear transcripts, affect tumor growth, invasion and metastasis, angiogenesis, immune escape, and stemness by regulating the expression of target genes in epigenetic, transcriptional, and posttranscriptional processes [7–9]. Abundant research has investigated the key effects of circRNAs on the initiation and development of malignant tumors, especially the latent clinical significance of circRNAs in the therapeutic targets and diagnostic biomarkers of CRC [10–13]. Accumulative studies have shown that circRNAs acted as competing endogenous RNAs (ceRNAs) via sponging microRNAs to significantly influence tumor progression, including cell growth, metastasis, and chemosensitivity [10, 14, 15]. Hence, exploring and clarifying the mechanisms of ceRNAs in emerging circRNAs can provide a new target for CRC therapeutics.

In general, this study first validated that circTMEM59 was downregulated in CRC tissues and cell lines. In addition, reduced circTMEM59 was correlated with adverse clinical characteristics and worse prognosis of CRC patients. Through multiple functional experiments in vitro and in vivo, we demonstrated that elevated circTMEM59 suppressed the growth and metastasis of CRC cells and accelerated cell apoptosis. With further investigation, we found that circTMEM59 acted as ceRNAs by competitively binding to miR-668-3p to modulate the expression of the inhibitor of DNA binding 4 (ID4), thereby influencing CRC progression. To conclude, this study first clarified the antitumor effects of circTMEM59/miR-668-3p/ID4 axis on CRC and provided potential therapeutic targets and clinical prognostic markers for CRC.

## 2. Materials and Methods

### 2.1. Tissue Specimen and Cell Culture

In this study, 100 paired CRC tissues and corresponding adjacent normal tissues were collected from the Department of Colorectal Surgery, the First Affiliated Hospital of Nanjing Medical University (NMU). None of the enrolled CRC patients received chemotherapy, radiotherapy, or immunotherapy before the surgery. All samples were retained at -80°C. All patients signed informed consent before surgery. The study was approved by the Ethics Committee of the First Affiliated Hospital of NMU (Ethics No.: 2020-SR-431).

HT29, LoVo, HCT116, SW480, DLD1, and NCM460 were purchased from the Cell Bank of Type Culture Collection of the Chinese Academy of Sciences (Shanghai, China). HT29 was cultured in McCoy's 5A medium (Thermo Fisher, Waltham, USA) supplemented with 10% fetal bovine serum (FBS) (Winsent, Quebec, Canada). RPMI 1640 (Thermo Fisher, Waltham, USA) supplemented with 10% FBS was used to culture the DLD1 cells. The cell culture mediums were supplemented with penicillin (100 U/ml) and streptomycin (100 *μ*g/ml). All cells were placed at 37°C in an incubator containing a 5% concentration of CO_2_.

### 2.2. RNA Isolation, qRT-PCR, and RT-PCR for microRNAs

The nuclear and cytoplasmic RNAs were isolated with a PARIS™ kit (Thermo Fisher, Waltham, USA). The specific procedures of qRT-PCR and RT-PCR for microRNAs were consistent with the previous publication [16]. The primers for qRT-PCR of circRNA, mRNAs, microRNA, and internal control (U6 and *β*-actin) and primers for RT-PCR of microRNA are listed in Supplementary Table 1. The relative expression of genes was presented by using the 2^−*ΔΔ*CT^ method.

### 2.3. Cell Transfection

*Lentivirus* of sh-circTMEM59, circTMEM59, miR-668-3p mimics, miR-668-3p inhibitor, and corresponding negative control (NC) were designed and synthesized by GenePharma (Shanghai, China). GenePharma also constructed pcDNA3.1 vectors containing ID4, sh-ID4, and the matched NC. The cells transfections were performed at 30%–40% confluence. Puromycin and neomycin were used to sift the stable transfected cells.

### 2.4. Cell Proliferation Assays

The specific procedures of Cell Counting Kit-8 (CCK-8), colony formation assay, and 5-ethynyl-2′-deoxyuridine (EdU) were conducted as previously described [17]. The appropriate wavelength (450 nm) was chosen to detect the cell absorbance after adding CCK-8 reagents into cells for 2 hours in the CCK-8 assay. For colony formation assay, crystal violet was used to stain cell colonies after 600 CRC cells were cultivated in six-well plates for 10 days. The images were photographed by Nikon fluorescence microscope after all the experimental procedures were completed according to the manufacturers' instructions in the EdU assay.

### 2.5. Transwell Assay

Specific procedures were consistent with the previous description [16]. Transwell inserts without Matrigel coating were used to measure the cell migratory ability. After adding Matrigel coating into inserts, cell invasion ability can be investigated. In each group, five random fields were selected and counted using a Nikon microscope (Nikon, Japan).

### 2.6. Cell Cycle and Apoptosis Analysis

The cells digested with trypsin were washed twice with PBS. Then, cells were fixed in 75% ethanol and stored at -20°C for at least 12 hours. Afterward, 75% ethanol was removed, and the cells were washed twice with PBS. An appropriate amount of DNA staining dye (propidium iodide, PI) (Vanzyme, Nanjing, China) was added into cells for half an hour in the dark at room temperature.

H_2_O_2_ (4 *μ*mol) was added into cells for 2 hours to accelerate cell apoptosis before adding the apoptosis reagents (Annexin V-FITC and PI) (Vanzyme, Nanjing, China). BD FACSCanto II (BD Biosciences, San Jose, CA, USA) was used to analyze the cell cycle distribution and apoptotic rate of CRC cells.

### 2.7. Western Blot Analysis

The detailed procedures of western blot analysis were performed as previous description [16]. ECL (Millipore, Burlington, MA, USA) solution was used to display the level of protein expression by using the Bio-Imaging System. The details of primary antibodies used in the study are as follows: ki-67 (Abcam, ab15580), Bcl-2 (Abcam, ab32124), E-cadherin (Cell Signaling Technology, #14472), Bax (Proteintech, 50599-2-Ig), ID4 (Santa Cruz Biotechnology, sc-365656; Abcam, ab49261), caspase-3 (Cell Signaling Technology, #9662), CDK4 (Abcam, ab108357), Vimentin (Cell Signaling Technology, #5741), cyclin D1 (Abcam, ab 134175), and GAPDH (Abcam, ab ab9485).

### 2.8. Animal Models

BALB/c nude mice (male, 4 weeks old) were purchased from the Animal Core Facility of NMU (Nanjing, China). 1 × 10^6^ cells were subcutaneously injected on both armpits of each mouse. Then, the width and length of the tumor were measured and recorded every week. The mice were euthanized at 3rd week, and the tumors were striped, photographed, weighed, and fixed in formaldehyde in turn. Subsequently, these tumors were subjected to IHC and TUNEL detection.

Briefly, 10^6^ cells suspended in 20 *μ*L phosphate buffer saline were injected into the distal tip of the spleen of mice to construct the metastatic model of CRC cells. After injection, the spleen was removed. The liver tissues were dissected and fixed in formaldehyde for hematoxylin and eosin (H&E) staining 4 weeks later. The animal experiment was ratified by the Animal Ethics Committee of NMU.

### 2.9. Immunohistochemistry (IHC)

The specific methods of IHC were consistent with the previous description [16]. IHC scores were composed of two parts (staining intensity and positive percentage). The staining intensity was divided into four levels, namely, 0, 1, 2, and 3, corresponding to undyed, weak, medium, and strong staining, respectively. Similarly, positive percentage were divided into five levels, namely, 0, 1, 2, 3, and 4, corresponding to 0%–5%, 6%–25%, 26%–50%, 51%–75%, and 76%–100% positive staining cells, respectively. Five random fields were chosen to obtain the average IHC score.

### 2.10. Fluorescent In Situ Hybridization (FISH)

The detailed steps were in accordance with those performed in a previous publication [18]. CircTMEM59-specific Cy3-labeled probe was used to detect the subcellular localization of circTMEM59 in CRC cells by using a FISH Kit (RiboBio, Guangzhou, China). The subcellular localization of miR-668-3p in CRC cells was detected by miR-668-3p detection probe (GenePharma, Shanghai, China).

### 2.11. Luciferase Reporter Assay

The sequences matched the 3′-UTR of circTMEM59 and ID4 mRNA, and those containing the wild-type or mutant miR-668-3p binding sites were designed and synthesized by GenScript (Nanjing, China). Procedures were conducted as the previous description [16].

### 2.12. RNA Immunoprecipitation (RIP)

The RIP Kit (Millipore, Burlington, MA, USA) was used to conduct the RIP assays according to the manufacturer's instructions. We extracted the coimmunoprecipitated RNA from the precipitates for further qRT-PCR. RIP assay was performed as previous description [17].

### 2.13. TUNEL Assay

TUNEL assay was performed as previous description [19]. The nucleus of the cell was stained by DAPI. The fluorescence microscope (Leica, Germany) was used to obtain images, which were then randomly filed.

### 2.14. Statistical Analysis

Each experiment was conducted independently thrice in this study. SPSS 22.0 (Chicago, USA) and GraphPad Prism 8.0 (CA, USA) software were used for the processing and analyzing data. Chi-square test, *t*-test, two-way ANOVA, Pearson correlation analysis, and Kaplan-Meier analysis were performed based on the experimental requirements. In each analysis, the significance threshold was set to 0.05. The data were shown as mean ± standard deviation.

## 3. Results

### 3.1. CircTMEM59 Is Downregulated in CRC Tissues and Cell Lines and Correlates with Adverse Clinical Characteristics

**Figure 1:**
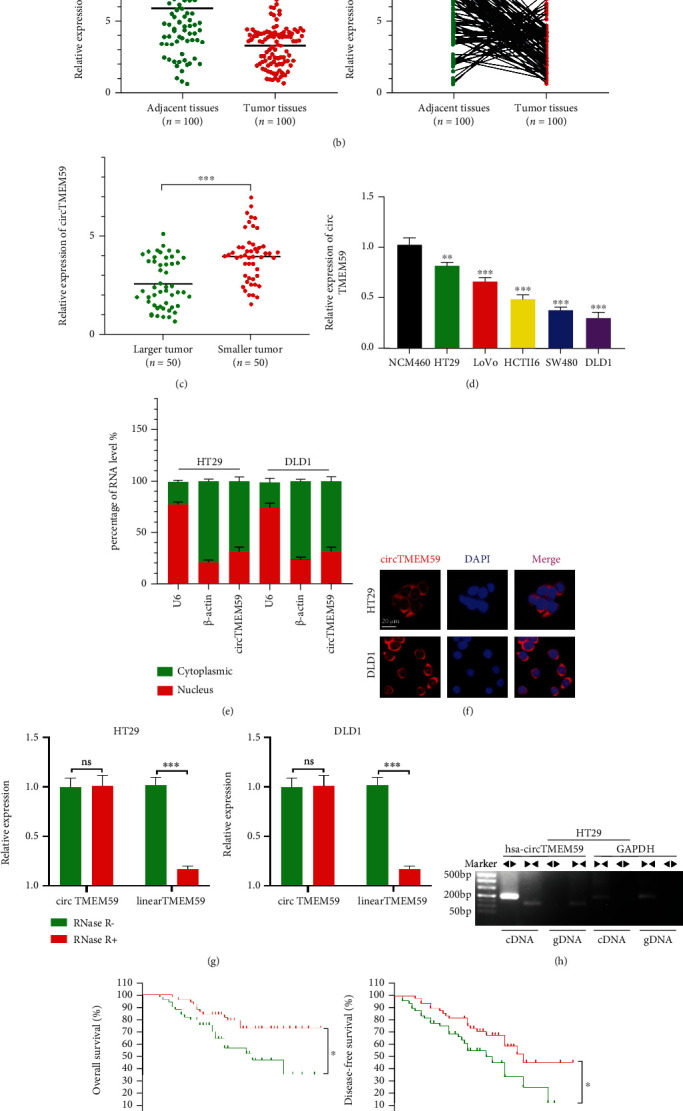
CircTMEM59 validation and expression in CRC cells and tissues. (a) Volcano plot analysis of circTMEM59 expression in CRC from the GEO database (GSE142837). (b) circTMEM59 expression in 100 paired CRC tissues and adjacent normal tissues. (c) circTMEM59 expression in different tumor size of CRC. (d) CircTMEM59 expression in CRC cell lines and NCM460 cell lines. (e, f) Subcellular localization of circTMEM59 in CRC cells was analyzed by qRT-PCR and FISH (scale bar: 20 *μ*m for FISH assay). (g) CircTMEM59 and linear TMEM59 expression levels were detected after RNase R treatment in CRC cells. (h) PCR and agarose gel electrophoresis confirmed the circular formation of circTMEM59, using divergent and convergent primers in gDNA and cDNA of HT29. GAPDH was used as a negative control. (i) Kaplan-Meier analysis of overall survival (OS) and disease-free survival (DFS) of CRC patients according to circTMEM59 expression levels. (j) The IHC of ki-67 and ID4 in the adjacent tissues, high expression circTMEM59 of tumors, and low expression circTMEM59 of tumors (scale bar: 100 *μ*m). Data represent the mean ± SD. Student's *t*-test was used to determine statistical significance: ^ns^*p* > 0.05, ∗*p* < 0.05, ∗∗*p* < 0.01, and ∗∗∗*p* < 0.001.

To investigate the key circRNAs involved in CRC advancement, the GEO database (GSE142837) was employed to explore the differential expression of circRNAs in CRC tissues compared with adjacent tissues. The expression of circTMEM59 (circBase ID: hsa_circ_0012634) was remarkably decreased in CRC tissues than in adjacent samples (Figure 1(a)). Furthermore, downregulated cireTMEM59 was confirmed in the matched 100 CRC tissues and 100 adjacent normal tissues (Figure 1(b)). We divided patients into two groups (larger and smaller tumor groups) according to the length of the CRC tumors. As shown in Figure 1(c), circTMEM59 was higher in smaller tumors than in larger tumors. Moreover, the results of circTMEM59 in CRC cell lines and NCM460 indicated that circTMEM59 expression in normal colorectal mucosa epithelial cells was higher than in the five CRC cell lines (Figure 1(d)). In addition, qRT-PCR and FISH assays confirmed that circTMEM59 was largely located in the cytoplasm of CRC cells (Figures 1(e) and 1(f)). RNase R was added into the CRC cells to authenticate the circular structure of circTMEM59, and qRT-PCR showed that circTMEM59 was more resistant to RNase R than linearTMEM59 mRNA in CRC cells (Figure 1(g)). Besides, the results of gel electrophoresis presented that the convergent primers of circTMEM59 and GAPDH could amplify the expected size products from cDNA and genomic DNA (gDNA). Only the divergent primers of circTMEM59 could amplify the PCR products from cDNA but not from gDNA. The divergent primers of GAPDH could not amplify the PCR products from cDNA or gDNA (Figure 1(h)). The abovementioned results showed that circTMEM59's structure was circular rather than linear. In addition, the relationship between the abundance of circTMEM59 and clinical characteristics and prognosis was explored via the collection and analysis of the data from 100 CRC patients. The data showed that the patients with lower expression of circTMEM59 had a worse prognosis (OS and DFS) than patients with higher expression (Figure 1(i)). Furthermore, circTMEM59 expression was significantly and negatively correlated with tumor size, TNM staging system, tumor stage, and the metastasis of surrounding lymph node (Table 1). It revealed that aberrant circTMEM59 was closely related to the proliferation and metastasis of CRC. Apart from that, the results of IHC also indicated that tumors with low expression of circTMEM59 grew faster and expressed lower levels of ID4 in comparison with tumors with high expression of circTMEM59 or the adjacent tissues (Figure 1(j)). The abovementioned facts suggested that circTMEM59 was downregulated in CRC; it can serve as clinical biomarkers for CRC.

### 3.2. CircTMEM59 Represses Cell Cycle Transition and Facilitates Cell Apoptosis and Metastasis of CRC In Vitro

**Figure 2:**
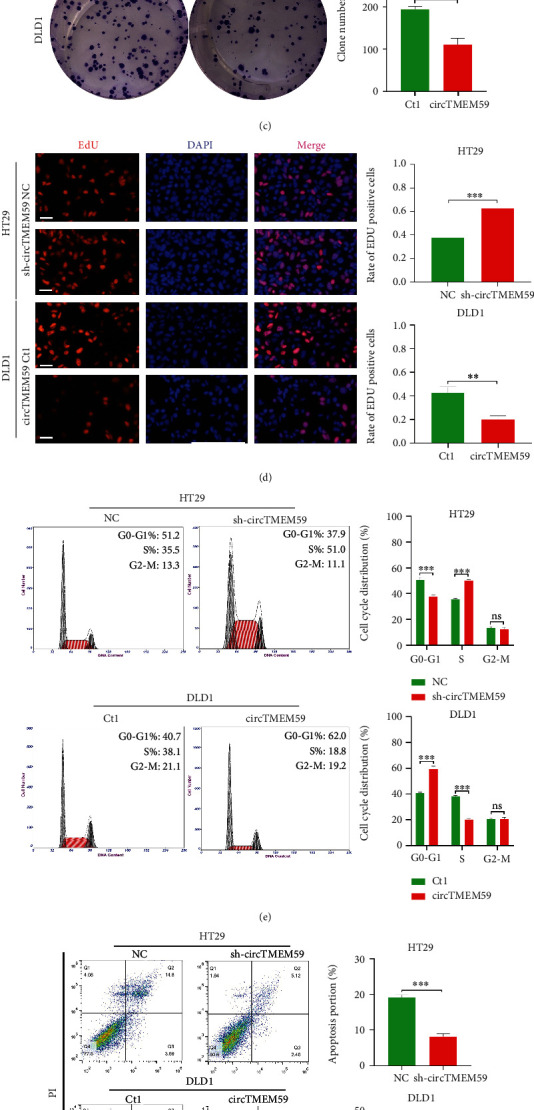
Overexpressed circTMEM59 inhibits cell proliferation and metastasis of CRC in vitro. (a) qRT-PCR analysis of circTMEM59 in HT29 treated with sh-circTMEM59 and DLD1 cells treated with LV-circTMEM59. (b) Effects of downregulated and upregulated circTMEM59 on proliferation in CRC cell lines were detected by CCK-8 assay. (c) Colony formation assays were conducted to measure cell proliferation ability in HT29 and DLD1 cells. (d) Cell proliferation of CRC cells measured by EDU staining. Scale bar = 100 *μ*m. (e) Effects of circTMEM59 on regulating cell cycle were detected by flow cytometry. (f) The apoptotic rates were detected by flow cytometry in circTMEM59 knockdown or overexpression cells. (g) The migration and invasion abilities were detected by Transwell assays in circTMEM59 knockdown or overexpression cells (scale bar: 100 *μ*m). (h) The IHC of ID4, Bcl-2, caspase-3, CDK4, cyclin D1, E-cadherin, and Vimentin in the low expression circTMEM59 of tumors and high expression circTMEM59 of tumors (scale bar: 100 *μ*m). Data represent the mean ± SD. Two-way ANOVA and Student's *t*-test was used to determine statistical significance: ^ns^*p* > 0.05, ∗∗*p* < 0.01, and ∗∗∗*p* < 0.001.

HT29 and DLD1 cell lines were chosen to conduct the functional experiments of circTMEM59 via the different abundance of circTMEM59 in CRC cell lines. As shown in Figure 2(a), qRT-PCR affirmed that circTMEM59 was downregulated in HT29 cells and overexpressed in DLD1 cells. Downregulated circTMEM59 promoted the cell growth of CRC via CCK-8 assay, whereas elevated circTMEM59 significantly repressed the cell proliferation of CRC (Figure 2(b)). Similarly, the results of colony formation assay and EdU assay also verified that the reduction of circTMEM59 facilitated cell proliferation, and the overexpression of circTMEM59 restrained the cell growth of CRC (Figures 2(c) and 2(d)). In addition, the overexpression of circTMEM59 arrested the cell cycle transition in the G0-G1 phrase, whereas reduction of circTMEM59 propelled the CRC cells transition to the S phrase (Figure 2(e)). Furthermore, increased circTMEM59 accelerated the cell apoptosis of CRC. However, apoptotic rates of HT29 were significantly decreased with the reduction of circTMEM59 (Figure 2(f)). Transwell assay demonstrated that migration and invasion abilities were greatly improved when circTMEM59 were downregulated in HT29 cells. On the contrary, overexpressed circTMEM59 dramatically suppressed migrating cell number and invasion of DLD1 (Figure 2(g)). The photographs of IHC showed that CRC tissues with a low level of circTMEM59 had higher expressions of Bcl-2, CDK4, Vimentin, and cyclin D1 and lower expressions of ID4, caspase-3, and E-cadherin compared with the high level of circTMEM59 expression in tumors of CRC (Figure 2(h) and Figure S1A). These data demonstrated that the overexpression of circTMEM59 prohibited CRC cells progression by preventing the cell cycle from entering the S phase, promoting cell apoptosis, and restraining cell metastasis in vitro.

### 3.3. CircTMEM59 Inhibits CRC Progression by Acting as a Molecular Sponge of miR-668-3p

**Figure 3:**
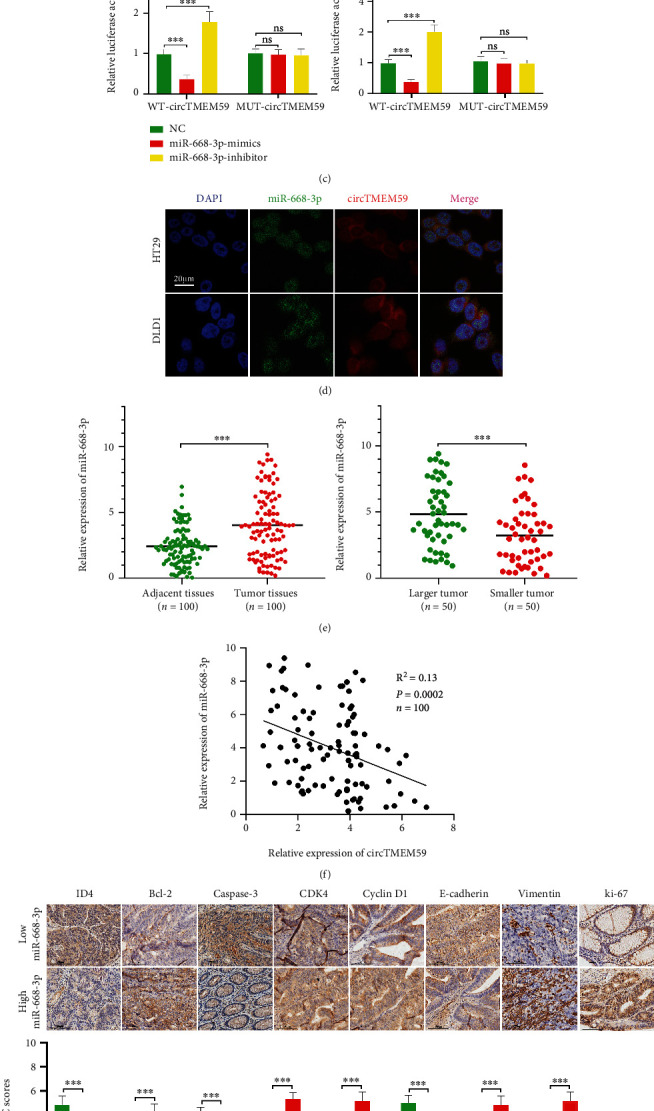
CircTMEM59 acts as a molecular sponge for miR-668-3p in CRC cells. (a) miRDB and circBank databases predict miRNAs binding to circTMEM59. (b) The predicted binding sites between circTMEM59 and miR-668-3p. (c) miR-668-3p mimics markedly reduced luciferase activity in circTMEM59-wild, not in circTMEM59-mut in HEK-293 T cells. (d) FISH assays showed that circTMEM59 and miR-668-3p colocalized in the cytoplasm of HT29 and DLD1 (scale bar: 20 *μ*m). (e) Relative expression of miR-668-3p in adjacent tissues, CRC tissues, and different tumor size of CRC. (f) Correlation analysis of the expression of circTMEM59 and miR-668-3p in 100 CRC tissues. (g) The IHC of ID4, Bcl-2, caspase-3, CDK4, cyclin D1, E-cadherin, Vimentin, and ki-67 in the low expression miR-668-3p of tumors and high expression miR-668-3p of tumors (scale bar: 100 *μ*m). Data represent the mean ± SD. Student's *t*-test was used to determine statistical significance: ^ns^*p* > 0.05 and ∗∗∗*p* < 0.001.

Previous research has reported that circRNAs located in the cytoplasm mainly exerted its effects on tumor progression by endogenously competing with microRNAs [10]. FISH and qRT-PCR had confirmed the cytoplasm location of circTMEM59. miRDB (http://mirdb.org/) and circBank (http://www.circbank.cn/index.html) were analyzed to seek the potential microRNAs that circTMEM59 bound to. Hsa-miR-668-3p was chosen from candidates that probably interacted with circTMEM59 through integrating and analyzing the screening data (Figure 3(a)). Then, a series of experiments were conducted to verify the hypothesis. First, overexpressed miR-668-3p significantly suppressed the luciferase activity of wild-type circTMEM59 and reduced miR-668-3p elevated the luciferase activity of wild-type circTMEM59 in CRC cells, whereas no significance was detected in the cells of mutant circTMEM59, as shown by luciferase reporter assay (Figures 3(b) and 3(c)). Second, the FISH assay validated the colocation of circTMEM59 and miR-668-3p in the cytoplasm of CRC cells (Figure 3(d)). Third, the expression of miR-668-3p was remarkably increased in CRC tissues than in adjacent samples (Figure 3(e)). Moreover, miR-668-3p increased in bigger tumors than that in smaller tumors (Figure 3(e)). In addition, miR-668-3p expression was significantly and positively correlated with tumor size, TNM staging system, tumor stage, and metastasis of the surrounding lymph nodes in CRC patients (Table 1). The expression of miR-668-3p was significantly and negatively correlated with the level of circTMEM59 expression in 100 CRC specimens (Figure 3(f)). The results of IHC showed that the CRC tissues with high miR-668-3p presented more proliferation and metastasis-related proteins (upregulated CDK4, cyclin D1, ki-67, and Vimentin and downregulated ID4 and E-cadherin) and less apoptosis-related proteins (upregulated Bcl-2 and downregulated caspase-3) than CRC tissues with low miR-668-3p (Figure 3(g)). Collectively, these data revealed that a competing endogenous RNA (ceRNA) relationship existed between circTMEM59 and miR-668-3p in CRC.

### 3.4. circTMEM59 Exerts Its Effects on CRC Cells by Regulating miR-668-3p

**Figure 4:**
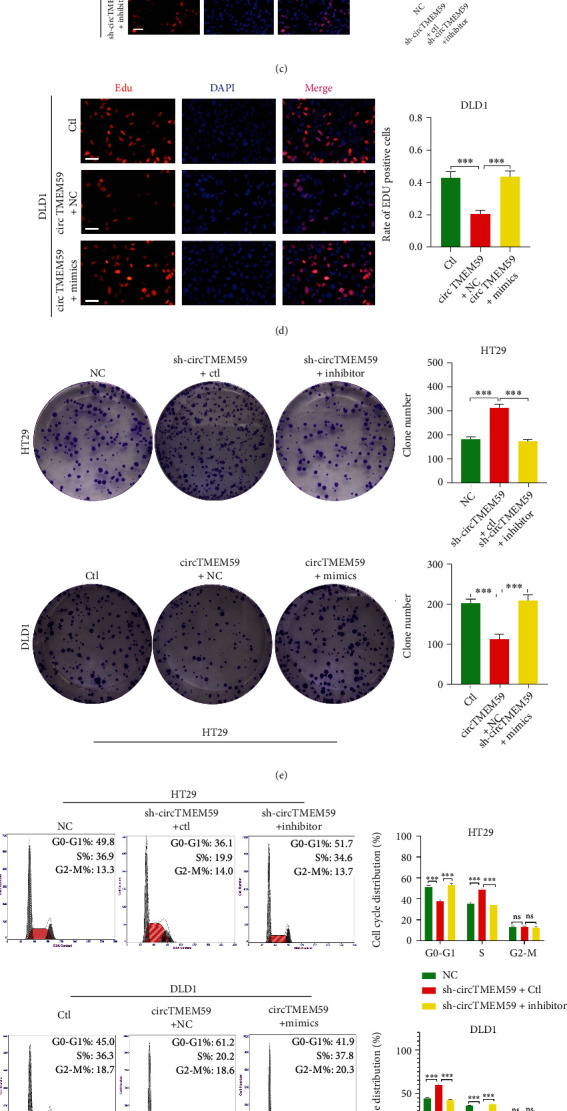
The effects of circTMEM59 on CRC cells are mediated by miR-668-3p. (a) Expression of miR-668-3p was verified in cotransfected CRC cell lines by qRT-PCR. (b–e) CCK-8 assays, EdU assays, and colony formation assays confirmed that miR-668-3p mediated the effects of circTMEM59 on proliferation of CRC cells (scale bar: 100 *μ*m for EdU assay). (f) Effects of circTMEM59 and miR-668-3p on regulating cell cycle in cotransfected CRC cell lines. (g) Knockdown miR-668-3p facilitated cell apoptosis of circTMEM59-downregulating of HT29 cells, while miR-668-3p restoration inhibited cell apoptosis of circTMEM59-overexpressing of DLD1 cells. (h) The migration and invasion abilities were detected by Transwell assays in cotransfected CRC cell lines (scale bar: 100 *μ*m). Data represent the mean ± SD. Student's *t*-test was used to determine statistical significance: ^ns^*p* > 0.05, ∗∗*p* < 0.01, and ∗∗∗*p* < 0.001.

A series of rescue experiments were conducted to investigate the biological role of miR-668-3p interacting with circTMEM59 in CRC. In HT29 cells, cotransfected miR-668-3p-inhibitor repressed the sh-circTMEM59-induced miR-668-3p elevation. In DLD1, the expression of miR-668-3p was reversed in the cotransfected miR-668-3p-mimics cells compared with the upregulated circTMEM59 group (Figure 4(a)). Downregulated miR-668-3p restrained the promotion of cell growth by circTMEM59 reduction, whereas overexpressed miR-668-3p abolished the repressed effects of elevated circTMEM59 on cell growth of CRC, as determined by CCK-8, EdU, and colony formation (Figures 4(b)–4(e)). As shown in Figure 4(f), decreased miR-668-3p expression abolished the effects of circTMEM59 reduction on cell cycle promotion to the S phrase, and increased miR-668-3p expression reverted the effects of circTMEM59 overexpression on cell cycle arrest in the G0–G1 phrase. Similar results were observed in the apoptosis assay in the cotransfected cells, decreased miR-668-3p expression abolished the effects of circTMEM59 reduction on cell apoptosis inhibition, and increased miR-668-3p expression reverted the effects of circTMEM59 overexpression on cell apoptosis acceleration (Figure 4(g)). In addition, the migration and invasion cell number decreased in the cotransfected sh-circTMEM59 + miR-668-3p-inhibitor HT29 cells compared with the cotransfected sh-circTMEM59 + Ctl HT29 cells, whereas the metastatic cell number increased in the cotransfected circTMEM59 + miR-668-3p-mimics DLD1 cells compared with the cotransfected circTMEM59 + NC DLD1 cells (Figure 4(h)). These data suggested that the suppression of CRC cell proliferation and metastasis by circTMEM59 is regulated by miR-668-3p.

### 3.5. CircTMEM59 Represses Cell Proliferation and Metastasis of CRC In Vivo

**Figure 5:**
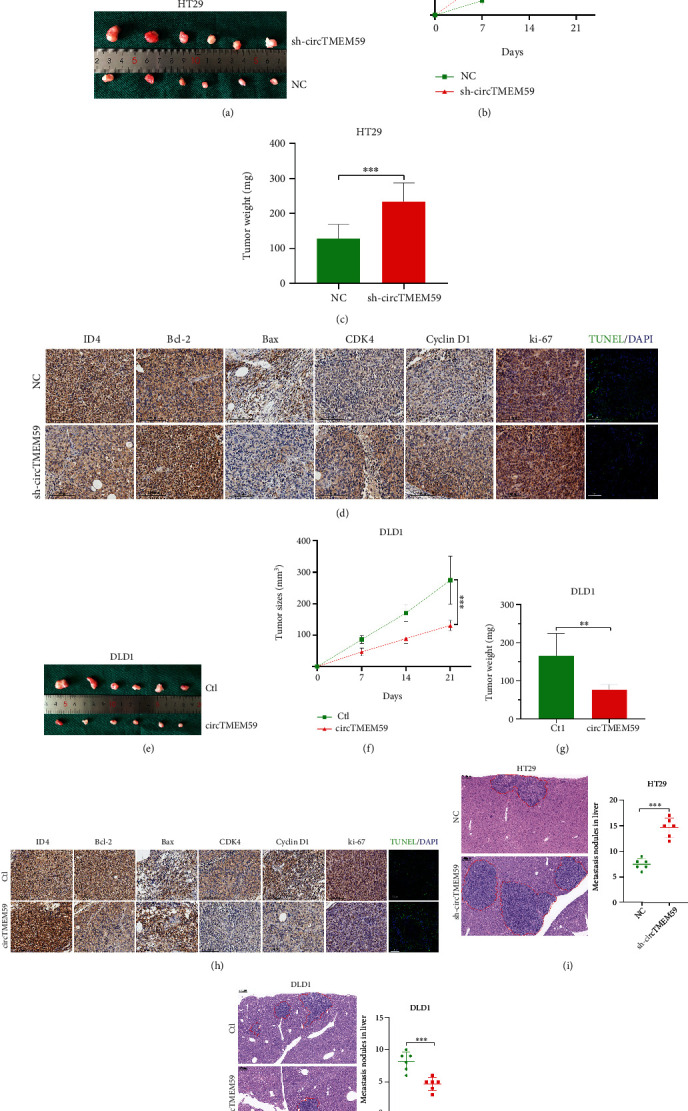
CircTMEM59 inhibits tumorigenicity and metastasis of CRC cells in vivo. (a) Photograph of subcutaneous tumors obtained from nude mice injected with HT29 cells (sh-circTMEM59 and NC). (b) Tumors were observed and recorded by tumor size. (c) Analysis of tumor weight in different groups. (d) Protein levels of ID4, Bcl-2, Bax, CDK4, cyclin D1, and ki-67 in the tumor samples of different groups were determined by IHC. Scale bar = 100 *μ*m. TUNEL was used to detect the apoptosis in the tumor samples. Scale bar = 50 *μ*m. (e) Photograph of subcutaneous tumors obtained from nude mice injected with DLD1 cells (Ctl and circTMEM59). (f) Tumors were observed and recorded by tumor size. (g) Analysis of tumor weight in corresponding groups. (h) Protein levels of ID4, Bcl-2, Bax, CDK4, cyclin D1, and ki-67 in the tumor samples of corresponding groups were determined by IHC (scale bar: 100 *μ*m). TUNEL was used to detect the apoptosis in the tumor samples. Scale bar = 50 *μ*m. (i, j) Representative HE staining of liver metastatic tumors. Scale bar = 200 *μ*m. Data represent the mean ± SD. Two-way ANOVA and Student's *t*-test were used to determine statistical significance: ∗∗*p* < 0.01 and ∗∗∗*p* < 0.001.

To investigate the biological functions of circTMEM59 in vivo, we chose a subcutaneous mouse model that is more easily and accurately monitored, although the orthotopic mouse model can better provide the anatomical site and microenvironment of CRC [20]. Decreased circTMEM59 HT29 cells (HT29-sh-circTMEM59) and negative control cells (HT29-NC) or elevated circTMEM59 DLD1 cells (DLD1-circTMEM59) and negative control cells (DLD1-Ctl) were subcutaneously injected into the nude mice. As shown in Figure 5(a), downregulated circTMEM59 facilitated the tumor proliferation of CRC cells. Furthermore, the tumors of the sh-circTMEM59 group grew much larger and heavier compared with those in the NC group (Figures 5(b) and 5(c)). Through the TUNEL and IHC assays, downregulated circTMEM59 promoted cell proliferation and inhibited cell apoptosis by increasing Bcl-2, CDK4, cyclin D1, and ki-67 expressions and decreasing ID4, Bax expressions, and TUNEL-positive cells (Figure 5(d) and Figure S1B). Conversely, upregulated circTMEM59 significantly suppressed the tumor growth of CRC cells (Figure 5(e)). In addition, the tumors in the DLD1-circTMEM59 group were much smaller and lighter than those in the DLD1-Ctl group (Figures 5(f) and 5(g)). Upregulated circTMEM59 resulted in tumors with less proliferation-related proteins (decreased CDK4, cyclin D1, ki-67, and increased ID4) and more apoptosis-related proteins (increased Bax and TUNEL-positive cells and decreased Bcl-2) (Figure 5(h) and Figure S1C). The circTMEM59 knockdown group showed the most number of and largest foci in the liver of nude mice (Figure 5(i)). Inversely, overexpressed circTMEM59 in DLD1 cells led to a massive decrease in size of foci and number of liver metastasis nodules (Figures 5(j)). The abovementioned data presented that circTMEM59 suppressed the proliferative and metastatic activities of CRC and regulated the level of proliferation-related proteins, apoptosis-related proteins, and ID4 expression in vivo.

### 3.6. ID4, a Target of miR-668-3p, Is Indirectly Modulated by circTMEM59

**Figure 6:**
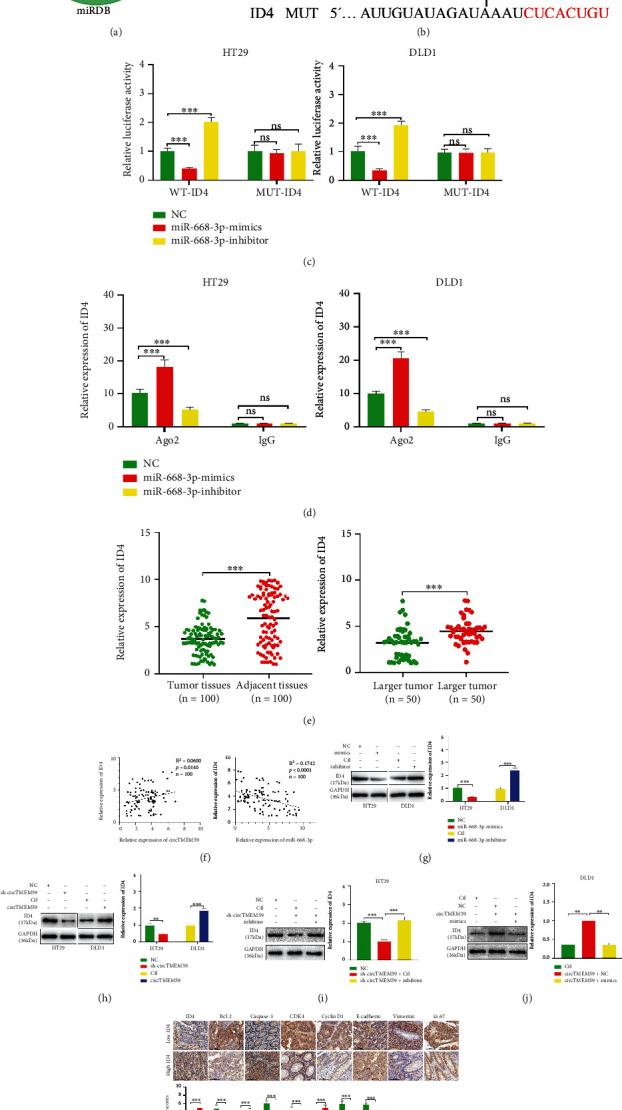
ID4, a target gene of miR-668-3p, is regulated by circTMEM59. (a) Venn diagram of predicted miR-668-3p targets by three programs (miRWalk, TargetScan, and miRDB). (b) The seed regions of miR-668-3p, the seed-recognizing sites in the ID4 3′ UTR, and the nucleotides mutated in ID4 mutant 3′ UTR are shown. (c) Luciferase reporter assay was conducted to verify that miR-668-3p bound to the 3′-UTR region of ID4 directly. miR-668-3p overexpression significantly suppressed, while miR-668-3p loss increased the luciferase activity that carried wild-type (WT) but not mutant (MUT) 3′-UTR of ID4. (d) RIP assays confirmed the binding status between miR-668-3p and ID4 in CRC cell lines, respectively. (e) Relative expression of ID4 in CRC tissues, adjacent tissues, and different tumor size of CRC. (f) Correlation analysis of the expression of circTMEM59 and ID4, miR-668-3p and ID4 in 100 CRC samples. (g) miR-668-3p overexpression decreased the level of ID4 protein in HT29 cells, while miR-668-3p reduction increased the level of ID4 protein in DLD1 cells. (h) CircTMEM59 reduction decreased the level of ID4 protein in HT29 cells, and circTMEM59 overexpression increased the level of ID4 protein in DLD1 cells. (i) Relative protein levels of ID4 in HT29 cells cotransfected with NC, sh-circTMEM59 + Ctl, and sh-circTMEM59 + inhibitor. (j) Relative protein levels of ID4 in DLD1 cells cotransfected with Ctl, circTMEM59 + NC, and circTMEM59 + mimics. (k) The IHC of ID4, Bcl-2, caspase-3, CDK4, cyclin D1, E-cadherin, Vimentin, and ki-67 in the low expression ID4 of tumors and high expression ID4 of tumors (scale bar: 100 *μ*m). Data represent the mean ± SD. Student's *t*-test was used to determine statistical significance: ^ns^*p* > 0.05, ∗∗*p* < 0.01, and ∗∗∗*p* < 0.001.

To further investigate the ceRNA network among circTMEM59, miR-668-3p, and downstream targets in CRC, miRWalk (http://mirwalk.umm.uni-heidelberg.de/), miRDB (http://mirdb.org/), and TargetScan (http://www.targetscan.org/vert_72/) databases were employed to explore the direct targets of miR-668-3p (Figure 6(a)). As presented in Figure 6(b), a conserved site of 3′-UTR of ID4 mRNA was identified to be directly bound to miR-668-3p. Then, luciferase reporter assay (Figure 6(c)) and RIP assay (Figure 6(d)) were conducted to validate the hypothesis that miR-668-3p directly binds to 3′-UTR of ID4 mRNA. The expression of ID4 was measured in the 100 paired CRC tissues and adjacent normal tissues by qRT-PCR. As expected, ID4 was simultaneously decreased in CRC tissues and larger tissues compared with that in adjacent tissues and smaller tissues (Figure 6(e)). Pearson's regression analysis revealed that the ID4 expression was a positive correlation with circTMEM59 expression, whereas it was a negative correlation with miR-668-3p expression in 100 CRC tissues (Figure 6(f)). Thereafter, immunoblot assays validated that elevated miR-668-3p decreased the expressions of ID4 protein in the CRC cells, whereas reduced miR-668-3p increased them (Figure 6(g)). Reduced circTMEM59 decreased the expressions of ID4 protein in the CRC cells, and elevated circTMEM59 increased them (Figure 6(h)). Furthermore, downregulated miR-668-3p reversed the reduction of ID4 protein resulting from reduced circTMEM59 (Figure 6(i)). Overexpressed miR-668-3p inverted the elevation of ID4 expression caused by elevated circTMEM59 (Figure 6(j)). IHC results showed that the CRC tissues with low ID4 expression presented more proliferation and metastasis-related proteins (upregulated CDK4, cyclin D1, ki-67, and Vimentin and downregulated E-cadherin) and less apoptosis-related proteins (upregulated Bcl-2 and downregulated Caspase-3) than CRC tissues with high ID4 expression (Figure 6(k)). These data demonstrated that circTMEM59 repressed cell growth and metastasis and facilitated cell death through competing binding to miR-668-3p to accumulate ID4 expression of CRC.

### 3.7. circTMEM59 Exerts Its Effects on CRC Cells by Indirectly Regulating ID4

**Figure 7:**
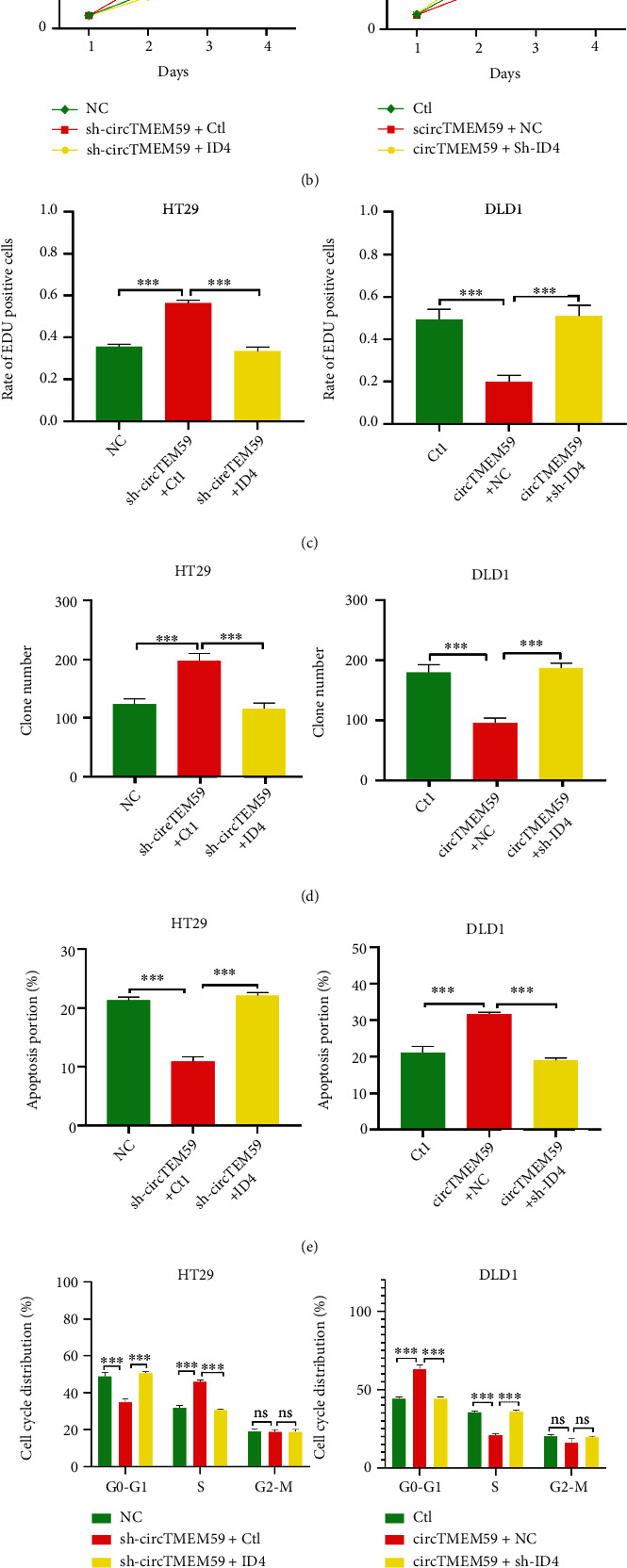
The regulation of circTMEM59 on CRC cells is mediated by ID4. (a) Expression of ID4 was confirmed by western blot in cotransfected CRC cell lines. (b–d) CCK-8 assays, EdU assays, and colony formation assays confirmed that ID4 mediated the effects of circTMEM59 on proliferation of CRC cells. (e) ID4 overexpression facilitated cell apoptosis of circTMEM59-downregulating of HT29 cells, while ID4 reduction inhibited cell apoptosis of circTMEM59-overexpressing of DLD1 cells. (f) Effects of circTMEM59 and ID4 on regulating cell cycle in cotransfected CRC cell lines. (g) The migration and invasion abilities were detected by Transwell assays in cotransfected CRC cell lines. (h) Western blot analysis confirmed that circTMEM59 inhibits cell cycle transition and cell metastasis and promotes cell apoptosis through regulating miR-668-3p of CRC. (i) Immunoblot analysis indicated that circTMEM59 restrains cell cycle transition and cell metastasis and facilitates cell apoptosis through regulating ID4 via sponging miR-668-3p of CRC. Data represent the mean ± SD. Two-way ANOVA and Student's *t*-test were used to determine statistical significance: ^ns^*p* > 0.05, ∗∗*p* < 0.01, and ∗∗∗*p* < 0.001.

**Figure 8:**
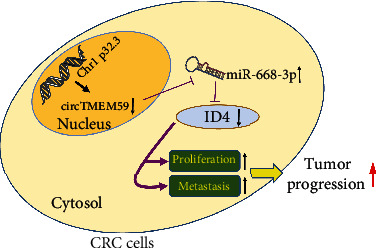
Schematic model of circTMEM59 suppresses proliferation and metastasis by acting as ceRNA of miR-668-3p to modulate ID4 in CRC cells.

Previous research has reported that ID4 acts as a tumor suppressor in CRC progression [21, 22]. We conducted the rescue experiments to confirm the interaction between circTMEM59 and ID4 of CRC. Immunoblot assays validated that elevated ID4 reversed the reduction of ID4 protein caused by reduced circTMEM59 (Figure 7(a)). In addition, downregulated ID4 inverted the ID4 increment, which resulted from elevated circTMEM59 (Figure 7(a)). Moreover, upregulated ID4 restrained the promotion of cell growth by circTMEM59 reduction, whereas reduced ID4 abolished the repressive effects of elevated circTMEM59 on CRC cell growth, as shown in CCK-8, EdU, and colony formation assays (Figures 7(b)–7(d), Figures S1D and S1E). Similar results were observed in the apoptosis assay, in which the cotransfected cells that upregulated ID4 abolished the effects of circTMEM59 reduction on cell apoptosis inhibition, and the reduced ID4 reverted the effects of circTMEM59 overexpression on cell apoptosis acceleration (Figure 7(e), Figure S1F). As presented in Figure 7(f) and Figure S1G, upregulated ID4 abolished the effects of circTMEM59 reduction on cell cycle promotion, and the reduced ID4 reverted the effects of circTMEM59 overexpression on cell cycle arrest. In addition, the migration and invasion cell number decreased in the cotransfected sh-circTMEM59 + ID4 HT29 cells compared with the cotransfected sh-circTMEM59 + Ctl HT29 cells, whereas the metastatic cell number increased in the cotransfected circTMEM59 + sh-ID4 DLD1 cells compared with the cotransfected circTMEM59 + NC DLD1 cells (Figure 7(g), Figure S1H). Western blot results demonstrated that downregulated miR-668-3p abolished the effects of circTMEM59 reduction on the elevation of Bcl-2, CDK4, cyclin D1, and Vimentin and the depletion of cleaved caspase-3 and E-cadherin. Upregulated miR-668-3p reverted the effects of circTMEM59 overexpression on the depletion of Bcl-2, CDK4, cyclin D1, and Vimentin and the elevation of cleaved caspase-3 and E-cadherin (Figure 7(h)). Furthermore, upregulated ID4 abolished the effects of circTMEM59 reduction on the elevation of Bcl-2, CDK4, cyclin D1, and Vimentin and the depletion of cleaved caspase-3 and E-cadherin. Downregulated ID4 reverted the effects of circTMEM59 overexpression on the depletion of Bcl-2, CDK4, cyclin D1, and Vimentin and the elevation of cleaved caspase-3 and E-cadherin (Figure 7(i)). To conclude, the abovementioned results confirmed that upregulated circTMEM59 inhibits cell growth and metastasis by miR-668-3p/ID4 axis in CRC (Figure 8).

## 4. Discussion

CircRNAs, which are emerging noncoding RNAs with loop structure, are widely reported in connection with the malignant progression of carcinoma, including sustaining growth, avoiding immune destruction, activating metastasis, and reprograming metabolism [11, 14, 23–25]. Reactive oxygen species (ROS) are natural byproducts of oxidative phosphorylation on the electron transport chain in mitochondria. The imbalance between ROS production and degradation can cause oxidative stress, resulting in energy depletion, DNA damage, protein carbonylation, and lipid oxidation, leading to various diseases and disorders [26, 27]. The close relationship between circular RNAs and ROS suggests that circular RNAs play critical roles as a target in the treatment of tumor metabolism [28–30]. Focusing on circRNAs as emerging molecular propellants of CRC progression, we intended to determine the mechanisms underlying circular RNA-mediated development of CRC. In this study, circTMEM59 was first explored in CRC progression as a tumor suppressor. CircTMEM59's downregulated expression in CRC tissues and cell lines was verified (Figure 1). The aberrant profiles of noncoding RNAs in the tumor tissues or the circulation can also be used to predict the clinicopathological characteristics and long-term survival of tumor patients [16, 31, 32]. In this study, CRC patients with high expression of circTMEM59 presented better clinicopathological characteristics (smaller tumor size, shallower tumor infiltration depth, and less lymph node metastasis) and longer survival time (Table 1 and Figure 1). These integral results showed that circTMEM59 acted as a tumor inhibitor in CRC and can be used as a clinical biomarker for diagnosis and prognosis.

The aberrant regulations of the cell cycle, cell apoptosis, and metastatic behaviors have played critical roles in the proliferation and progression of tumors [19, 31, 33, 34]. Some studies revealed that circRNAs play key roles in the regulation of the cell cycle, cell apoptosis, and distant metastases of CRC [11, 14, 15]. A series of functional assays validated that the upregulated circTMEM59 inhibited cell growth and cell metastasis of CRC, whereas downregulated circTMEM59 accelerated the cell cycle shift and metastatic behaviors and repressed apoptosis of CRC cells in vitro and in vivo (Figures 2 and 5). Combined with clinical data, IHC results showed that CRC tissues with a low level of circTMEM59 had higher expressions of Bcl-2, CDK4, cyclin D1, and Vimentin and lower expressions of ID4, caspase-3, and E-cadherin compared with the high level of circTMEM59 in CRC tissues (Figure 2). The abovementioned results explore the potentiality and orientation of manipulating circTMEM59 for CRC therapy, combined with the effective application of noncoding RNA-delivering systems [35].

Recent studies reported that circRNA functions as ceRNA of microRNAs in the cytoplasm to affect the tumor progression [10, 15]. qRT-PCR and FISH assays confirmed that circTMEM59 was largely located in the cytoplasm of CRC cells. miR-668-3p was identified as a target of circTMEM59 by bioinformatic tools and luciferase reporter assay, which is reported to be an oncogenic gene in hepatocellular carcinoma [36]. The miR-668-3p was elevated in 100 CRC tissues and positively correlated with adverse clinical features, according to the analysis of clinical specimens and data (Figure 3). Moreover, miR-668-3p was negative relation to circTMEM59 expression in 100 CRC tissues. IHC images presented that CRC tissues with high miR-668-3p presented more proliferation and metastasis-related proteins (upregulated CDK4, cyclin D1, ki-67, and Vimentin and downregulated ID4 and E-cadherin) and less apoptosis-related proteins (upregulated Bcl-2 and downregulated caspase-3) than CRC tissues with low miR-668-3p expression (Figure 3). In addition, miR-668-3p mediated the effects of circTMEM59 on the cell cycle, cell apoptosis, and metastasis of CRC. Decreased miR-668-3p expression abolished the effects of circTMEM59 reduction on cell proliferative and metastatic promotion and cell apoptosis inhibition. Increased miR-668-3p expression reverted the effects of circTMEM59 overexpression on cell proliferative suppression, metastatic inhibition, and cell apoptosis acceleration (Figure 4). The intrinsic ceRNA mechanisms between circTMEM59 and miR-668-3p were gradually revealed in CRC.

To further determine the detailed ceRNAs mechanisms of circTMEM59 in CRC, three datasets were employed to seek the key targets of miR-668-3p. ID4 was identified as a target of miR-668-3p through luciferase reporter and RIP assays, which was abnormal in different types of cancer [37–39]. Importantly, ID4 is reportedly an inhibitor of CRC progression [21, 22]. As expected, ID4 was simultaneously decreased in CRC tissues and larger tissues compared with that in adjacent tissues and smaller tissues. Moreover, ID4 was positively correlated with circTMEM59 and was negatively correlated with miR-668-3p in 100 CRC tissues (Figure 6). In addition, miR-668-3p restrained and circTMEM59 facilitated the abundance of ID4 proteins in CRC cells. Through the results of IHC, the CRC tissues with low ID4 expression presented more proliferation and metastasis-related proteins (upregulated CDK4, cyclin D1, ki-67, and Vimentin and downregulated E-cadherin) and less apoptosis-related proteins (upregulated Bcl-2 and downregulated caspase-3) than CRC tissues with high ID4 expression (Figure 6). Rescue experiments revealed that upregulated ID4 restrained the promotion in cell growth and metastasis of circTMEM59 reduction, whereas reduced ID4 abolished the repressed effects of increased circTMEM59 on cell growth, migration, and invasion of CRC (Figure 7). Taken together, all data suggested that elevated circTMEM59 prohibited cell cycle transition (CDK4 and cyclin D1), inhibited cell metastasis (E-cadherin and Vimentin) and promoted cell apoptosis (cleaved caspase-3 and Bcl-2) by accumulating ID4 by sponging for miR-668-3p in CRC (Figure 8).

## 5. Conclusion

In general, this study validated for the first time that circTMEM59 was downregulated in CRC tissues and cell lines. In addition, low circTMEM59 expression in CRC patients was closely related to shorter survival time and advanced clinicopathological characteristics. Furthermore, circTMEM59 repressed the cell growth and metastasis and accelerated cell apoptosis of CRC via accumulating ID4 by sponging miR-668-3p. In conclusion, the aberrant circTMEM59 had profound effects on tumor growth, migration, and invasion and was an effective biomarker and therapeutic target for CRC.

## Figures and Tables

**Table 1:** Relevance analysis of circTMEM59 and miR-668-3p expression in CRC patients.

Variable	CircTMEM59		*p* value	miR-668-3p		*p* value
	High	Low		High	Low	
All cases	50	50		50	50	
Age (years)						
<60	22	21	0.8395	20	23	0.5446
≥60	28	29		30	27	
Gender						
Male	28	27	0.8415	26	29	0.5463
Female	22	23		24	21	
Tumor size (cm)						
<4	35	19	0.0013^∗∗^	20	34	0.0050^∗∗^
≥4	15	31		30	16	
TNM staging system						
T1 + T2	34	21	0.0090^∗∗^	22	33	0.0270^∗^
T3 + T4	16	29		28	17	
Tumor stage						
Stage I + II	33	18	0.0027^∗∗^	20	31	0.0278^∗^
Stage III + IV	17	32		30	19	
Lymph node metastasis						
No	36	21	0.0025^∗∗^	23	34	0.0263^∗^
Yes	14	29		27	16	
Distant metastasis						
No	42	36	0.1475	38	40	0.6293
Yes	8	14		12	10	

∗*p* < 0.05 and ∗∗*p* < 0.01.

## Data Availability

The datasets used in the study are available from the corresponding author on reasonable request.

## Supplementary Materials

Supplementary Materials Supplementary Material Supplementary Figure 1: the regulation of circTMEM59 on CRC cells is mediated by ID4. A The IHC scores of ID4, Bcl-2, Caspase-3, CDK4, cyclin D1, E-cadherin, and Vimentin in the low expression circTMEM59 of tumors and high expression circTMEM59 of tumors. B The IHC scores of ID4, Bcl-2, Bax, CDK4, cyclin D1, and ki-67 and TUNEL data in NC group and sh-circTMEM59 group. C The IHC scores of ID4, Bcl-2, Bax, CDK4, cyclin D1, and ki-67 and TUNEL data in Ctl group and circTMEM59 group. D Effects of circTMEM59 and ID4 on proliferation in cotransfected CRC cell lines were detected by EdU assays (scale bar: 100 µm). E Effects of circTMEM59 and ID4 on proliferation in cotransfected CRC cell lines were detected by colony formation. F ID4 overexpression facilitated cell apoptosis of circTMEM59-downregulating of HT29 cells, while ID4 reduction inhibited cell apoptosis of circTMEM59-overexpressing of DLD1 cells. G Effects of circTMEM59 and ID4 on regulating cell cycle in cotransfected CRC cell lines. H The migration and invasion abilities were detected by Transwell assays in cotransfected CRC cell lines (scale bar: 100 µm). Supplementary Table 1: the primers for qRT-PCR and RT-PCR.

## References

[1] Sung H., Ferlay J., Siegel R. L. (2021). Global cancer statistics 2020: GLOBOCAN estimates of incidence and mortality worldwide for 36 cancers in 185 countries. *CA: a Cancer Journal for Clinicians*.

[2] Cao W., Chen H. D., Yu Y. W., Li N., Chen W. Q. (2021). Changing profiles of cancer burden worldwide and in China: a secondary analysis of the global cancer statistics 2020. *Chinese Medical Journal*.

[3] Wolpin B. M., Mayer R. J. (2008). Systemic treatment of colorectal cancer. *Gastroenterology*.

[4] Saad E. D., Katz A., Hoff P. M., Buyse M. (2010). Progression-free survival as surrogate and as true end point: insights from the breast and colorectal cancer literature. *Annals of oncology: official journal of the European Society for Medical Oncology*.

[5] Shi J., Huang H., Guo L. (2015). Acceptance and willingness-to-pay for colorectal colonoscopy screening among high-risk populations for colorectal cancer in urban China. *Zhonghua yu fang yi xue za zhi [Chinese journal of preventive medicine]*.

[6] Normanno N., Cervantes A., Ciardiello F., De Luca A., Pinto C. (2018). The liquid biopsy in the management of colorectal cancer patients: Current applications and future scenarios. *Cancer treatment reviews*.

[7] Chen L. L. (2020). The expanding regulatory mechanisms and cellular functions of circular RNAs. *Nature Reviews. Molecular Cell Biology*.

[8] Ma Z., Shuai Y., Gao X., Wen X., Ji J. (2020). Circular RNAs in the tumour microenvironment. *Molecular Cancer*.

[9] Beilerli A., Gareev I., Beylerli O. (2021). Circular RNAs as biomarkers and therapeutic targets in cancer. *Seminars in Cancer Biology*.

[10] Zhou R., Jia W., Gao X. (2021). CircCDYL acts as a tumor suppressor in Wilms' tumor by targeting miR-145-5p. *Frontiers in cell and developmental biology*.

[11] Peng C., Tan Y., Yang P. (2021). Circ-GALNT16 restrains colorectal cancer progression by enhancing the SUMOylation of hnRNPK. *Journal of experimental & clinical cancer research: CR*.

[12] Xie M., Yu T., Jing X. (2020). Exosomal circSHKBP1 promotes gastric cancer progression via regulating the miR-582-3p/HUR/VEGF axis and suppressing HSP90 degradation. *Molecular Cancer*.

[13] Wei Y., Lu C., Zhou P. (2021). EIF4A3-induced circular RNA ASAP1 promotes tumorigenesis and temozolomide resistance of glioblastoma via NRAS/MEK1/ERK1-2 signaling. *Neuro-Oncology*.

[14] Wang Q., Shi L., Shi K. (2020). CircCSPP1 functions as a ceRNA to promote colorectal carcinoma cell EMT and liver metastasis by upregulating COL1A1. *Frontiers in Oncology*.

[15] Yong W., Zhuoqi X., Baocheng W., Dongsheng Z., Chuan Z., Yueming S. (2018). Hsa_circ_0071589 promotes carcinogenesis via the miR-600/EZH2 axis in colorectal cancer. *Biomedicine & pharmacotherapy = Biomedecine & pharmacotherapie*.

[16] Li J., Peng W., Yang P. (2020). MicroRNA-1224-5p inhibits metastasis and epithelial-mesenchymal transition in colorectal cancer by targeting SP1-mediated NF-*κ*B signaling pathways. *Frontiers in Oncology*.

[17] Zhang Z., Li J., Huang Y. (2018). Upregulated miR-1258 regulates cell cycle and inhibits cell proliferation by directly targeting E2F8 in CRC. *Cell Proliferation*.

[18] Yang P., Li J., Peng C. (2020). TCONS_00012883 promotes proliferation and metastasis via DDX3/YY1/MMP1/PI3K-AKT axis in colorectal cancer. *Clinical and Translational Medicine*.

[19] Li J., Yang P., Chen F. (2021). Hypoxic colorectal cancer-derived extracellular vesicles deliver microRNA-361-3p to facilitate cell proliferation by targeting TRAF3 via the noncanonical NF-*κ*B pathways. *Clinical and Translational Medicine*.

[20] Mittal V. K., Bhullar J. S., Jayant K. (2015). Animal models of human colorectal cancer: current status, uses and limitations. *World Journal of Gastroenterology*.

[21] Chen H. J., Yu Y., Sun Y. X. (2021). Id4 suppresses the growth and invasion of colorectal cancer HCT116 cells through CK18-related inhibition of AKT and EMT signaling. *Journal of Oncology*.

[22] Umetani N., Takeuchi H., Fujimoto A., Shinozaki M., Bilchik A. J., Hoon D. S. B. (2004). Epigenetic inactivation of ID4 in colorectal carcinomas correlates with poor differentiation and unfavorable prognosis. *Clinical cancer research : an official journal of the American Association for Cancer Research*.

[23] Zhang P. F., Pei X., Li K. S. (2019). Circular RNA circFGFR1 promotes progression and anti-PD-1 resistance by sponging miR-381-3p in non-small cell lung cancer cells. *Molecular Cancer*.

[24] Liu Y., Ma L., Hua F. (2022). Exosomal circCARM1 from spheroids reprograms cell metabolism by regulating PFKFB2 in breast cancer. *Oncogene*.

[25] Li Q., Pan X., Zhu D., Deng Z., Jiang R., Wang X. (2019). Circular RNA MAT2B promotes glycolysis and malignancy of hepatocellular carcinoma through the miR-338-3p/PKM2 Axis under hypoxic stress. *Hepatology (Baltimore, Md.)*.

[26] Yang B., Chen Y., Shi J. (2019). Reactive oxygen species (ROS)-based nanomedicine. *Chemical Reviews*.

[27] Sena L. A., Chandel N. S. (2012). Physiological roles of mitochondrial reactive oxygen species. *Molecular Cell*.

[28] Zhang H., Ge Z., Wang Z., Gao Y., Wang Y., Qu X. (2021). Circular RNA RHOT1 promotes progression and inhibits ferroptosis via mir-106a-5p/STAT3 axis in breast cancer. *Aging*.

[29] Geng J., Yang K. (2021). circCCND1 regulates oxidative stress and FGF9 to enhance chemoresistance of non-small cell lung cancer via sponging miR-187-3p. *DNA and Cell Biology*.

[30] Yang C., Wu S., Mou Z. (2022). Exosome-derived circTRPS1 promotes malignant phenotype and CD8+ T cell exhaustion in bladder cancer microenvironments. *Molecular therapy: the journal of the American Society of Gene Therapy*.

[31] Li J., Zhang Z., Chen F. (2019). The diverse oncogenic and tumor suppressor roles of microRNA-105 in cancer. *Frontiers in Oncology*.

[32] Li J., Feng Y., Heng D. (2020). Circulating non-coding RNA cluster predicted the tumorigenesis and development of colorectal carcinoma. *Aging*.

[33] Shu F., Yang T., Zhang X. (2021). Hyaluronic acid modified covalent organic polymers for efficient targeted and oxygen-evolved phototherapy. *Journal of Nanobiotechnology*.

[34] Li J., Han X., Gu Y. (2021). LncRNA MTX2-6 suppresses cell proliferation by acting as ceRNA of miR-574-5p to accumulate SMAD4 in esophageal squamous cell carcinoma. *Frontiers in cell and developmental biology*.

[35] Singh A., Trivedi P., Jain N. K. (2018). Advances in siRNA delivery in cancer therapy. *Artificial cells, nanomedicine, and biotechnology*.

[36] Ma H., Huang C., Huang Q. (2021). Circular RNA circ_0014717 suppresses hepatocellular carcinoma tumorigenesis through regulating miR-668-3p/BTG2 Axis. *Frontiers in Oncology*.

[37] Cheng D., Fan J., Ma Y. (2019). LncRNA SNHG7 promotes pancreatic cancer proliferation through ID4 by sponging miR-342-3p. *Cell & Bioscience*.

[38] Donzelli S., Sacconi A., Turco C. (2020). Paracrine signaling from breast cancer cells causes activation of ID4 expression in tumor-associated macrophages. *Cell*.

[39] Gao X.-Z., Zhao W.-G., Wang G.-N., Cui M.-Y., Zhang Y.-R., Li W.-C. (2016). Inhibitor of DNA binding 4 functions as a tumor suppressor and is targetable by 5-aza-2'-deoxycytosine with potential therapeutic significance in Burkitt’s lymphoma. *Molecular Medicine Reports*.

