# Supplementary material for: Upregulated circTMEM59 Inhibits Cell Growth and Metastasis by miR-668-3p/ID4 Axis in Colorectal Cancer
Source: Oxid Med Cell Longev. 2022 May 24;2022:7242124. doi: 10.1155/2022/7242124 (PMC9155906; doi:10.1155/2022/7242124)
Supplement: Supplementary Materials — Supplementary Material Supplementary Figure 1: the regulation of circTMEM59 on CRC cells is mediated by ID4. A The IHC scores of ID4, Bcl-2, Caspase-3, CDK4, cyclin D1, E-cadherin, and Vimentin in the low expression circTMEM59 of tumors and high expression circTMEM59 of tumors. B The IHC scores of ID4, Bcl-2, Bax, CDK4, cyclin D1, and ki-67 and TUNEL data in NC group and sh-circTMEM59 group. C The IHC scores of ID4, Bcl-2, Bax, CDK4, cyclin D1, and ki-67 and TUNEL data in Ctl group and circTMEM59 group. D Effects of circTMEM59 and ID4 on proliferation in cotransfected CRC cell lines were detected by EdU assays (scale bar: 100 µm). E Effects of circTMEM59 and ID4 on proliferation in cotransfected CRC cell lines were detected by colony formation. F ID4 overexpression facilitated cell apoptosis of circTMEM59-downregulating of HT29 cells, while ID4 reduction inhibited cell apoptosis of circTMEM59-overexpressing of DLD1 cells. G Effects of circTMEM59 and ID4 on regulating cell cycle in cotransfected CRC cell lines. H The migration and invasion abilities were detected by Transwell assays in cotransfected CRC cell lines (scale bar: 100 µm). Supplementary Table 1: the primers for qRT-PCR and RT-PCR. [file 7242124.f1.docx]

**Upregulated circTMEM59 inhibits cell growth and metastasis by miR-668-3p/ID4 axis in colorectal cancer**

**Supplementary information**

**Supplementary Figure 1** The regulation of circTMEM59 on CRC cells is mediated by ID4. **A** The IHC scores of ID4, Bcl-2, Caspase-3, CDK4, Cyclin D1, E-cadherin and Vimentin in the low expression circTMEM59 of tumors and high expression circTMEM59 of tumors. **B** The IHC scores of ID4, Bcl-2, Bax, CDK4, Cyclin D1, and ki-67 and TUNEL data in NC group and sh-circTMEM59 group. **C** The IHC scores of ID4, Bcl-2, Bax, CDK4, Cyclin D1, and ki-67 and TUNEL data in Ctl group and circTMEM59 group. **D** Effects of circTMEM59 and ID4 on proliferation in co-transfected CRC cell lines were detected by EdU assays (scale bar: 100 μm). **E** Effects of circTMEM59 and ID4 on proliferation in co-transfected CRC cell lines were detected by colony formation. **F** ID4 overexpression facilitated cell apoptosis of circTMEM59-downregulating of HT29 cells, while ID4 reduction inhibited cell apoptosis of circTMEM59-overexpressing of DLD1 cells. **G** Effects of circTMEM59 and ID4 on regulating cell cycle in co-transfected CRC cell lines. **H** The migration and invasion abilities were detected by Transwell assays in co-transfected CRC cell lines (scale bar: 100 μm).

**Supplementary Table 1** The primers for qRT-PCR and RT-PCR.

**The primers for qRT-PCR.**

| hsa-circTMEM59 | Forward Primer | TGATGCCAAAAATGCACCTA |
| --- | --- | --- |
|  | Reverse Primer | ATTTGTAGGCTCCTGCTCCA |
| hsa-β-actin | Forward Primer | CATGTACGTTGCTATCCAGGC |
|  | Reverse Primer | CTCCTTAATGTCACGCACGAT |
| hsa-U6 | Forward Primer | CTCGCTTCGGCAGCACA |
|  | Reverse Primer | AACGCTTCACGAATTTGCGT |
| hsa-ID4 | Forward Primer | TCCCGCCCAACAAGAAAGTC |
|  | Reverse Primer | CCAGGATGTAGTCGATAACGTG |
| hsa-miR-668-3p | Forward Primer | CGGGCTGTCACTCGGCTCGGC |
|  | Reverse Primer | CAGCCACAAAAGAGCACAAT |
| hsa-GAPDH | Forward Primer | GGAGCGAGATCCCTCCAAAAT |
|  | Reverse Primer | GGCTGTTGTCATACTTCTCATGG |

**The primers for RT-PCR**

| hsa-miR-668-3p | CCTGTTGTCTCCAGCCACAAAAGAGCACAATATTTCAGGAGACAACAGGGTAGTGG |
| --- | --- |
| hsa-U6 | AACGCTTCACGAATTTGCGT |
